# The Effect of Low-Dose Atropine on Alpha Ganglion Cell Signaling in the Mouse Retina

**DOI:** 10.3389/fncel.2021.664491

**Published:** 2021-05-05

**Authors:** Qin Wang, Seema Banerjee, ChungHim So, ChunTing Qiu, YingHon Sze, Thomas Chuen Lam, Chi-Ho To, Feng Pan

**Affiliations:** ^1^School of Optometry, The Hong Kong Polytechnic University, Kowloon, Hong Kong; ^2^Centre for Eye and Vision Research, Hong Kong, Hong Kong

**Keywords:** atropine, retinal ganglion cells (RGCs), myopia, retina, vision, circuit

## Abstract

Low-dose atropine helps to control myopia progression with few side effects. However, the impact of atropine, a non-selective muscarinic Acetylcholine (ACh) receptor antagonist, on retinal ganglion cells (RGCs) remains unclear. After immersing the cornea and adjacent conjunctiva of enucleated eyes in 0.05% (approximately 800 μM) atropine solution for 30 min, the atropine concentration reached in the retina was below 2 μM. After direct superfusion of the retina with 1 μM atropine (considering that the clinical application of 0.05% atropine eye drops will be diluted over time due to tear flow for 30 min), no noticeable changes in the morphology of ON and OFF alpha RGCs (αRGCs) were observed. Atropine affected the light-evoked responses of ON and OFF αRGCs in a dose- and time-dependent fashion. Direct application of less than 100 μM atropine on the retina did not affect light-evoked responses. The time latency of light-induced responses of ON or OFF αRGCs did not change after the application of 0.05–100 μM atropine for 5 min. However, 50 μM atropine extended the threshold of joint inter-spike interval (ISI) distribution of the RGCs. These results indicated that low-dose atropine (<0.5 μM; equal to 1% atropine topical application) did not interfere with spike frequency, the pattern of synchronized firing between OFF αRGCs, or the threshold of joint ISI distribution of αRGCs. The application of atropine unmasked inhibition to induce ON responses from certain OFF RGCs, possibly *via* the GABAergic pathway, potentially affecting visual information processing.

## Introduction

Myopia is a significant public health problem because high myopia increases the risk of severe eye diseases, including retinal detachment, glaucoma, and cataract. Myopia is a leading cause of blindness (Shih et al., 2006; Holden et al., 2014; Russo et al., 2014). It has been predicted that 50% of the world’s population will be myopic by 2050 (Holden et al., 2016). Myopia currently affects over 80% of adults in Hong Kong and 22% of the global population (Vitale et al., 2008; Foster and Jiang, 2014). Despite its significant public health impact, myopia is arguably neither prevented nor reduced by any optical approaches to correct image defocus (Cooper and Tkatchenko, 2018).

Low-dose atropine is an effective therapy to control myopia progression. In children, atropine was widely used to prevent myopia progression to high myopia (Luu et al., 2005; Cooper and Tkatchenko, 2018; Wu et al., 2019). Our previous research revealed that defocused images changed multineuronal firing patterns and signaling of retinal ganglion cells (RGCs) in the mouse retina (Pan, 2019; Banerjee et al., 2020). ON delayed ganglion cells have been shown to play a role in myopia development (Mani and Schwartz, 2017). However, the impact of atropine on the signaling of RGCs remains unclear. This study aimed to determine the effects of atropine on these cells even if the lack of effects of atropine observed in this study can help clarify its action in myopia control by showing its minimal effects on retinal signaling.

Acetylcholine (ACh) is a primary retinal neurotransmitter that modulates visual processing through muscarinic and nicotinic acetylcholine receptors expressed on bipolar cells (Elgueta et al., 2015), amacrine cells (Keyser et al., 2000), and ganglion cells (Lipton et al., 1987; Reed et al., 2002; Marritt et al., 2005; Dmitrieva et al., 2007; Strang et al., 2010). ACh is only released by the gamma-aminobutyric acid (GABAergic)/cholinergic starburst amacrine cells (Voigt, 1986; Brecha et al., 1988; O’Malley and Masland, 1989). The light-evoked responses of RGCs are affected *via* muscarinic and/or nicotinic receptors preceding RGC activation (Reed et al., 2002; Strang et al., 2015).

Atropine is a broad, nonselective muscarinic acetylcholine receptor antagonist that has shown clinical effectiveness in myopia control. In animal experiments, atropine was shown to inhibit myopia induction in chicks whose striated ciliary muscle was innervated by the nicotinic receptor, not muscarinic receptors (Schmid and Wildsoet, 2004). Atropine can also prevent myopia induction in monkeys *via* muscarinic receptors (Tigges et al., 1999). Therefore, low-dose topical atropine might control myopia development through muscarinic receptors in the retina, not *via* the accommodation system. However, the effect of low-dose atropine on retinal signal transmission is not fully understood. Atropine sulfate (monohydrate) is the most widely used atropine compound. Low-dose atropine (0.01–0.05%) has been used for myopia control in clinical trials (Prousali et al., 2019). Atropine sulfate reaches the retina *via* transcorneal or transconjunctival–scleral pathways. For example, at 24 h after uniocular installation of 1% atropine eye drop, the concentration of atropine in the rabbit’s vitreous humor was approximately 0.4 μM (Wang et al., 2019). In the study, the atropine concentration reached the mouse retina after the external ocular application was determined by using targeted mass spectrometry.

Alpha retinal ganglion cells (αRGCs), which are present in all mammalian species, are characterized by large somas, broad dendritic fields and regularly spaced in the retina in a mosaic-like fashion across the species (Peichl, 1991; Zhang et al., 2005). αRGCs play an essential role in visual processing in the retina and transfer coded visual information to the brain (Krieger et al., 2017). The study of αRGCs can reflect and characterize the biophysical properties of all RGCs. This study evaluated the effects of topical (conjunctival) administration of 0.05–500 μM atropine on αRGCs, a range that covered doses administered to humans, although concentrations lower than 0.5 μM atropine did not alter the spike frequency, the synchronized firing pattern between OFF αRGCs, and the joint inter-spike interval (ISI) distribution threshold of RGCs. However, the application of 0.05 μM atropine induced the ON response in light-evoked responses of 25% OFF αRGCs. However, the ON response was not observed in OFF αRGCs in homozygous Cx36-knockout (KO) mice even after applying up to 500 μM atropine. The OFF αRGCs lost their heterozygous coupling with amacrine cells in Cx36 KO mice. Cx36 is required for OFF αRGCs coupled with amacrine cells to mediate crossover excitation in OFF αRGCs (Farajian et al., 2011). Therefore, atropine application might relate to unmasking inhibition of the GABAergic pathway.

In summary, low-dose atropine did not change the electrophysical properties of αRGCs. However, around one-fourth OFF αRGCs might unmask inhibition to induce ON response by interfering with the GABA pathway. This effect might disturb the accuracy of visual signaling transport to the central nervous system. The result indicates the caution about treating myopia with low-dose atropine.

### Materials and Methods

#### Animal Preparation

All experiments complied with the Guide for the Care and Use of Laboratory Animals published by the National Institutes of Health.

Adult mice (postnatal day 16–56) C57BL/6J (RRID: IMSR_JAX:000664) wild-type (WT) of either sex, weighing 15–20 g, were used in the study (*n* = 56). Kcng4-YFP (6–8 weeks, *n* = 11) mice (Duan et al., 2015), either sex, weighing 15–25 g, were used for the ON and OFF αRGCs labeling. Homozygous *Cx36* KO mice (RRID: MGI:3810172), first generated in the laboratory of David Paul, Harvard Medical School (Cambridge, MA, USA), were a kind gift from Samuel M. Wu, Baylor College of Medicine (*n* = 6, 6–8 weeks, weighing 15–25 g). All the animals were maintained in a 12-h light/12-h dark cycle. The mice were deeply anesthetized with an intraperitoneal injection of ketamine and xylazine (80 and 10 mg/kg body weight, respectively), and lidocaine hydrochloride (20 mg/ml) was applied locally to the eyelids and surrounding tissues before enucleation.

#### Flattened Retina Preparation

The eyes were removed under dim red illumination. The retinas were dissected into four equal quadrants for patch-clamp recordings and attached to a modified translucent Millicell filter ring (Millipore, Bedford, MA, USA). The flattened retinas were superfused with the oxygenated mammalian Ringer’s solution (Bloomfield and Miller, 1982). The bath solution was continuously bubbled with 95% O_2_–5% CO_2_ and maintained at approximately 32°C as described previously (Volgyi et al., 2013; Pan et al., 2016). The anesthetized animals were killed by cervical dislocation immediately after enucleation.

#### Immunohistochemistry Staining

The retinal pieces were fixed in 4% paraformaldehyde for at least 10 min before immunohistochemistry staining. The retina tissues were then incubated with choline acetyltransferase (ChAT, goat anti-ChAT, 1:500; Millipore; Cat# AB144P, RRID: AB_2079751) antibody for 3–7 days at 4°C, followed by incubation with the secondary antibodies overnight at 4°C. After washing with 0.1 M phosphate-buffered saline, the tissues were mounted for observation, and images were captured under a Zeiss LSM 800 with an Airyscan confocal microscope (ZEISS, Thornwood, NY, USA). A *z*-stack of the image was acquired at 0.35-μm steps at a resolution of 1,024 × 1,024 pixels. To measure the dendritic field equivalent diameter and soma size, each cell was measured from a two-dimensional projection. Maximum projection images were used for the determination of the dendritic field areas of the RGCs. A polygon was drawn by marking the dendrites’ edges in the image using Zen 2.3 microscope software (ZEISS Microscopy). Soma area was acquired by ImageJ software (ImageJ, an open-source program developed by the National Institutes of Health, Bethesda, MD, USA, 1.52i, RRID: nif-0000-30467), followed by calculating the polygon area, the equivalent diameter, and the major axis. The equation used for the measurement of the dendritic field equivalent diameter (Figure 2E) was $\text{2}\sqrt{\text{(A / π)}},$ where A is the polygon area. To compare the stratification of RGCs in the retinas (Figure 2), the dendrites of RGCs were acquired in confocal image stacks. Subsequently, the ChAT bands were used as a reference, and dendritic density was calculated relative to the inner plexiform layer (IPL) depth (Zhang et al., 2005).

**Figure 1:**
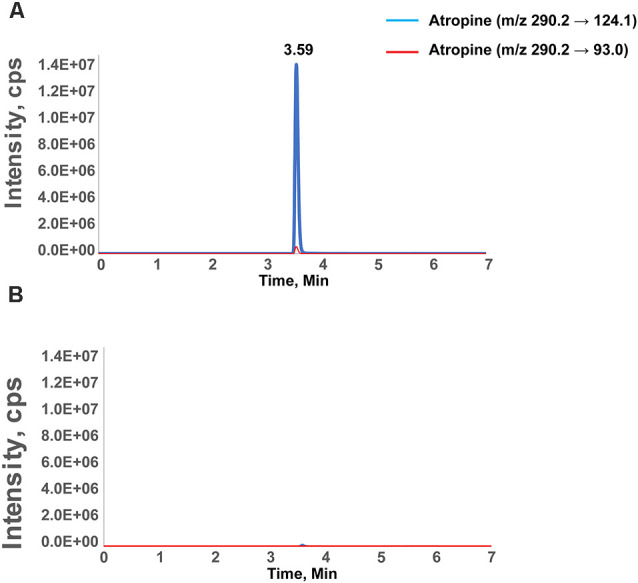
Chromatographic separation of atropine in the retina. Chromatographic separation of atropine in the retina after external ocular application of atropine **(A)** and the vehicle-treated contralateral eye **(B)**. The peak represents the identification of atropine as measured with two transitions and eluted at 3.59 min. The intensity is the signal readout from mass spectrometry. The transition peak area was integrated and quantified by calibration against retinal samples spiked with defined amounts of atropine. The X-axis is the retention time of the compound during chromatographic separation. The specific retention time and mass transitions increase the confidence level of identifying only the compound of interest (atropine, in this case). **(B)** A spectrum of the control retina, in which the signal below the limit of quantification (1 nM) results in a 10-fold signal-to-noise ratio.

**Figure 2:**
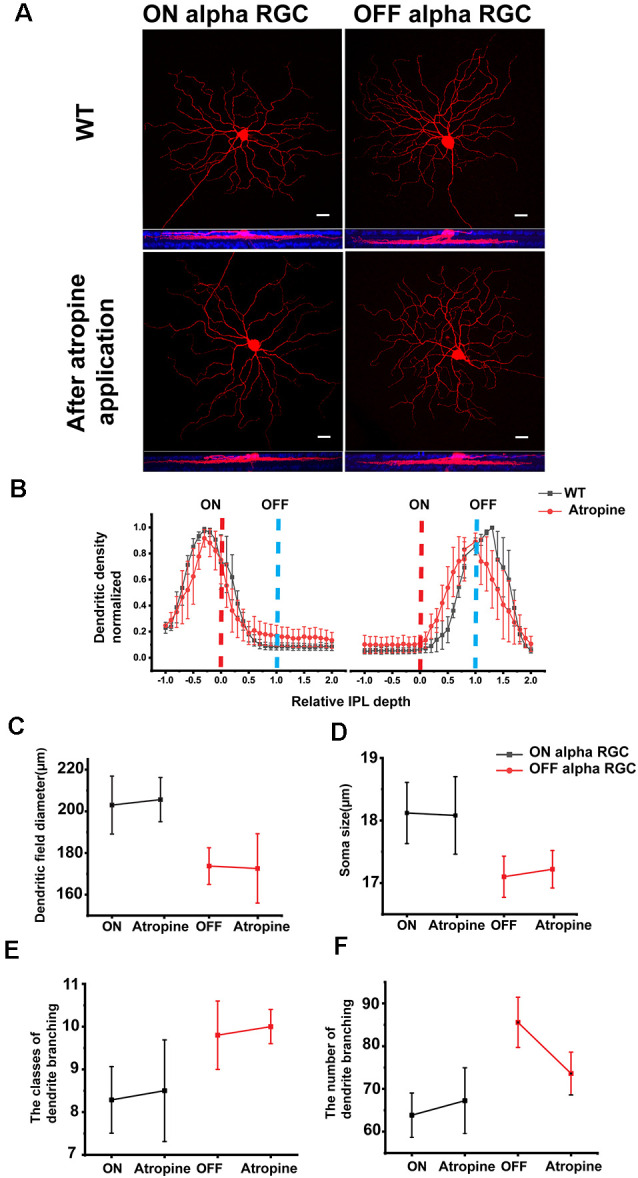
ON and OFF αRGCs in the retinas of wild-type mice before and after atropine application. Representative images of ON and OFF αRGCs in the mouse retina **(A)** show almost no morphological changes after 1 μM atropine for 30 min, compared with those cells in the wild-type (WT) mice. Images at the bottom show the ON and OFF αRGCs double-labeled with anti-ChAT antibody (blue). Scale bar = 20 μM. Stratification of ON and OFF αRGCs in WT and after atropine application **(B)**. Stratification was normalized with 0, and 1 corresponded to the ON and OFF ChAT labeled bands, respectively (dashed lines). The dendritic field equivalent diameter and somata size of ON and OFF αRGCs showed no statistical difference between the atropine-treated retinas and the WT mouse retinas **(C,D)**, and so with the classes and the number of dendrites branching of ON and OFF αRGCs **(E,F)**. The error bars from **(B)** to **(F)** represent SEM.

#### Light Stimulation

A green (λ = 525 nm) light-emitting diode (HLMP-CM3A-Z10DD, Broadcom Limited, San Jose, CA, USA) was used to deliver uniform full-field visual stimuli to the retinal surface. The intensity of the square wave light stimuli was calibrated and expressed in terms of the time-averaged rate of photoisomerization per rod per second (Rh* per rod/s; Pan et al., 2016). Recordings were performed in dark-adapted conditions.

#### Electrophysiology

Extracellular recordings were obtained from RGCs of the mid-peripheral retina in the nasotemporal plane. The recordings were performed using an Axopatch 700B amplifier connected to a Digidata 1550B interface and pCLAMP 10 software (Molecular Devices, San Jose, CA, USA). Cells were visualized with near-infrared light (>775 nm) at ×40 magnification with a Nuvicon tube camera (Dage-MTI, Michigan City, IN, USA) and differential interference optics on a fixed-stage microscope (Eclipse FN1; Nikon, Tokyo, Japan). The retinas were superfused at a rate of 1–1.5 ml min^−1^ with Ringer’s solution containing 120 mM NaCl, 2.5 mM KCl, 25 mM NaHCO_3_, 0.8 mM Na_2_HPO_4_, 0.1 mM NaH_2_PO_4_, 1 mM MgCl_2_, 2 mM CaCl_2_, and 5 mM D-glucose. The bath solution was continuously bubbled with 95% O_2_–5% CO_2_ at 32°C. Electrodes were pulled to 5−7 MΩ resistance, with the internal solution consisting of 120 mM potassium gluconate, 12 mM KCl, 1 mM MgCl_2_, 5 mM EGTA, 0.5 mM CaCl_2_, and 10 mM HEPES (adjusted to pH 7.4 with KOH). This internal solution was used in experiments to avoid the blockage of spiking.

Spike trains were recorded digitally at a sampling rate of 10 kHz with Axoscope software, which was sorted by an Offline Sorter (Plexon, Dallas, TX, USA) and NeuroExplorer (Nex Technologies, Littleton, MA, USA) software. For comparison purposes, the spike frequencies are normalized. The recorded cells were dye-injected with pipette tips filled with 4% Neurobiotin (Vector Laboratories, Burlingame, CA, USA) and 0.5% Lucifer Yellow-CH (Molecular Probes, Eugene, OR, USA) as previously described (Pan and Massey, 2007).

### Pharmacological Application of Atropine and GABA

Up to 500 μM atropine sulfate salt monohydrate (Sigma–Aldrich, St. Louis, MO, USA; A-0257, molecular weight 694.83) in Ringer’s solution was used for the electrical recordings of RGC.

Eight-hundred micromolar (equal to 0.05% atropine eye drop solution) was used for immersion of the enucleated eyes. To determine whether the external ocular application of atropine could reach the retina, the mouse eyeball was immersed into 800 μM atropine solution with the cornea side facing downward to the ciliary body level for 30 min. As a vehicle control, the contralateral eye was immersed in Ringer’s solution. Subsequently, the retinas of both groups were isolated for targeted mass spectrometry.

Then, 1 mM GABA was puffed using a Picospritzer III intracellular microinjection dispense system connected to a patch pipette (resistance, ~8–10 MΩ). Ringer’s solution was used as the puffing solution. The puff application of Ringer’s solution did not evoke detectable responses in RGCs.

### Standard Solution and Sample Preparation for Mass Spectrometry

A stock solution of 1 mM atropine in ethanol was diluted with 5% methanol and stored at −20°C. Working solutions of 100–500 nM atropine were freshly prepared from the stock and kept at 4°C before use. The sample preparation was similar to the metabolomics protocol, with few modifications for the retina samples (Paris et al., 2016; Yu et al., 2020). The retinas were placed in a 2-ml tube pre-packed with 1.4–2.8-mm ceramic beads in 200 μl methanol (CKMix, Precellys, Bertin Technologies, France), followed by homogenization at 5,800 rpm for four cycles of 30 s with 20-s intervals using a Precellys Evolution Tissue Homogenizer. The homogenate was transferred into a 1.5-ml Eppendorf tube and centrifuged at 15,000 rpm, 4°C, for 5 min. The supernatant was diluted 10-fold with deionized water and stored at −80°C before analysis.

### Liquid Chromatography–Tandem Mass Spectrometry Instrumentation

The sample was analyzed using an ultra-high-performance liquid chromatography (UHPLC) 110 system (Eksigent Technologies, Dublin, CA, USA) coupled with QTRAP 6500+ mass spectrometry (Sciex, Redwood City, CA, USA) equipped with Hypersil GOLD C18 (50 × 2.1 mm, 3 μM) HPLC column at 40°C. The samples were separated using a gradient of 0.1% formic acid in water (solution A) and 0.1% formic acid in methanol (solution B) at a flow rate of 0.5 ml per minute. The gradient was as follows: 5% solution B, held isocratic for 1 min; linear gradient to 95% over 3 min; equilibrated for 1 min and reconditioned at 5% solution B for 2 min for a total run time of 7 min. Samples (10 μl) were delivered in a partial loop fill injection mode and analyzed with electrospray ionization in positive ion mode. The interface conditions were as follows: curtain gas of 25; collision-activated dissociation at medium; IonSpray voltage at 5.5 kV; temperature of 300°C; source gas 1 of 35; and source gas 2 of 35. Atropine was monitored in the multiple reaction monitoring (MRM) mode. The precursor ion m/z 290.2 (close to positively ionized from 289.2 of atropine molecular weight) was optimized as follows: declustering potential at 106, entrance potential at 10 V, collision energy at 31 eV for the quantitative transition of m/z 290.2→124.1, 39 eV for the qualitative transition of m/z 290.2→93.0, and collision exit potential at 42 V. The peak finding and peak area integration were performed using the IntelliQuan algorithm (Sciex), and the concentrations of the liquid chromatography–tandem mass spectrometry data were calculated using the Analyst software (version 1.6.3, Sciex).

### Data Acquisition and Analysis

Statistical analyses were performed using Origin software (OriginLab, Northampton, MA, USA). Statistically significant differences (*P* < 0.05) were determined by Student’s *t*-tests. The result shown is mean value ± standard error of the mean (SEM), unless otherwise indicated.

### Results

#### Mass Spectrometry Detected Retinal Atropine After External Ocular Application

Targeted mass spectrometry is a powerful analytical technique to quantify defined substances within a sample. A mass spectrometer generates multiple ions when analyzing the sample under investigation. Subsequently, it separates them according to their specific mass-to-charge ratios, followed by recording the relative or absolute abundance of each ion type. This study used mass spectrometry to quantify atropine in the retina after external ocular application. Following targeted mass spectrometry, the peak representing atropine was measured with two transitions and eluted at 3.59 min (1,960.0 ± 524.2, mean ± SD, *n* = 5; Figure 1). This peak area was integrated and calibrated by adding a standard amount of atropine to retinal tissue in calibration runs. In addition, the spectra of the control retinas from the contralateral eye without atropine showed that the signal intensity readout was below the limit of quantification (1 nM; Table 1). Although the concentration of atropine in the retinas of treated eyes was approximately 400 times lower than at the ocular surface, i.e., it was approximately 2 μM in retinal tissue and approximately 800 μM in the (0.05% atropine) solution that was applied to the corneal side of the ocular surface. Targeted mass spectrometry using MRM confirmed that the external atropine application reached the retina, albeit in low concentration.

**Table 1:** The concentration of atropine in treated and control retinas (*n* = 5).

Sample	Retention time (min)	Ion ratio (93.0/124.1)	Concentration (nmol/L)	Recovery ratio (800 μM)	*P*-value
Retina from atropine-treated eyes	3.59 ± 0.01	0.0366 ± 0.0002	1,960.0 ± 524.2	408:1	0.00026
Control, retina from untreated contralateral eyes	3.59 ± 0.02	0.0365 ± 0.0019	<LOQ	-	-

*LOQ, limit of quantitation*.

Consistent retention time and ion ratio of atropine transitions confirmed the identification and accuracy. The limit of quantification at 1 nM atropine was set based on a 1-to-10 signal-to-noise ratio. Data are presented as mean ± SD. The recovery of atropine in the treated retinas was calculated as the mean value, estimated to be 426 times lower in the retina relative to the atropine solution applied externally on the corneal side of the ocular surface.

#### Atropine Had No Noticeable Effects on the Morphology of ON and OFF αRGCs

It had been shown previously that AChR-mediated retinal waves affect the development of RGC dendritic morphology and synaptic connection (Tian, 2011). Genetic deletion of β2 subunits of nicotinic acetylcholine receptor (nAChR) eliminated the retinal waves and disturbed the RGC dendritic stratification (Bansal et al., 2000). The axonal projections of α-type OFF RGC to the central nervous system are also impaired in β2 subunit KO mice (Cang et al., 2005; Huberman et al., 2008). Therefore, atropine may lead to the morphological change in ON and OFF αRGCs. To evaluate the effects of atropine on the morphology of αRGCs, 1 μM atropine was directly superfused onto the isolated retina for 30 min, and their detailed anatomic morphology was revealed by Neurobiotin to the ON and OFF αRGCs.

Furthermore, 2 μM atropine was detected in the mouse retina after applying 0.05% (i.e., 800 μM) atropine to the external ocular surface for 30 min. Considering the clinical application of 0.05% atropine, eye drops would get diluted over time due to tear flow. Thus, less than 1 μM atropine was used for the retina’s superfusion.

More than 40 subtypes of RGCs have been identified in the mouse retina (Volgyi et al., 2009; Pan et al., 2010; Baden et al., 2016). The ON and OFF αRGCs were selected to evaluate the atropine effects on cell morphology, confirmed with Neurobiotin injection.

ON αRGC and OFF αRGC cell groups were inspected following an individual injection with Neurobiotin before and after applying 1 μM atropine for 30 min (Figure 2A). Application of atropine did not affect the stratification nor was there any significant difference in the dendritic field diameter of either ON or OFF αRGCs (Figure 2B) ON αRGCs—before: 203 ± 13.9 μm, mean ± SEM, *n* = 6; after: 205.6 ± 10.6 μm, *n* = 4; *p* = 0.73; OFF αRGCs—before: 173.73 ± 8.8 μm, *n* = 4; after: 172.6 ± 16.6, *n* = 4; *p* = 0.91 (Figure 2C). Atropine application also had no effect on the soma diameter of either cell type (ON αRGCs—before: 18.12 ± 0.49 μm, *n* = 6; after: 18.08 ± 0.62 μm, *n* = 4; *p* = 0.97; OFF αRGCs—before: 17.1 ± 0.33 μm, *n* = 4; after: 17.22 ± 0.3 μm, *n* = 4; *p* = 0.79; Figure 2D). There were no statistically significant differences in the classes of dendrite branching of ON αRGCs (before: 8.3 ± 0.7, *n* = 6; after: 8.5 ± 1.2, *n* = 4; *p* = 0.89) and OFF αRGCs (before: 9.8 ± 0.8, *n* = 6; after: 10 ± 0.4, *n* = 4; *p* = 0.88; Figure 2E) after atropine application and so with the number of dendrites branching of ON αRGCs (before: 63.9 ± 5.2 μm, *n* = 6; after: 67.3 ± 7.7 μm, *n* = 4; *p* = 0.72) and OFF αRGCs (before: 85.6 ± 5.9 μm, *n* = 6; after: 73.6 ± 5 μm, *n* = 4; *p* = 0.15; Figure 2F).

Therefore, the application of 1 μM atropine for 30 min had no noticeable effects on the morphology of the ON and OFF αRGCs.

#### Atropine Dose-Dependently Inhibited Light-Evoked Responses in the ON and OFF αRGCs

Previous studies have shown that low-dose atropine slows down myopia progression (Wu et al., 2019). It has been suggested that retinal signaling in response to defocused images may be important in the development of myopia (Pan, 2019). Thus, this study aimed to determine whether the application of atropine affects the signaling of RGCs, i.e., the neuronal output of the retina to deliver visual information to the central nervous system. In this study, the light-evoked responses (spike frequencies) of the ON and OFF αRGCs were recorded following direct retinal exposure to increasing concentrations of atropine. Their spike frequencies were normalized from the same cells.

**Figure 3:**
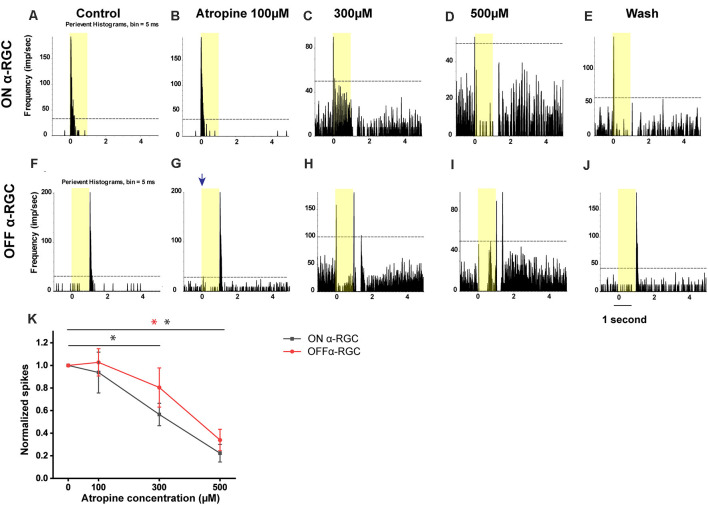
Dose-dependent effects of atropine application on light-evoked responses of ON and OFF αRGCs. Peristimulus time histograms (PSTHs) of light-evoked responses of ON αRGCs **(A)**. Presentation of the 525-nm full-field light stimulation; intensity = 131 Rh* per rod s^−1^ is indicated by the yellow bar. The dotted line represents 99% confidence level, above which the correlations are above chance. The light response of ON αRGC was shown after exposure to 100, 300, and 500 μM atropine (**B–D**, respectively). The spike frequency decreased with increased atropine concentration. The light response of ON αRGCs was recovered after 5 min of washing **(E)**. PSTHs of the light-evoked responses of OFF αRGCs to the same 525-nm full-field light stimulation **(F)**. Light responses of OFF αRGCs are shown after exposure to 100, 300, and 500 atropine (**G–I**, respectively). The spike frequency decreased at atropine concentrations of 300 and 500 μM. ON response was induced in the OFF αRGC after exposure to 100 μM atropine (blue arrow in **G**). The light response of OFF αRGC recovered after 5 min of washing **(J)**. A graph summarized the normalized spike frequency of ON and OFF αRGCs in response to increasing concentrations of atropine **(K)**. Then, 500 μM atropine significantly affected the spike frequencies of light-induced response in both ON and OFF αRGCs. Moreover, 300 μM atropine had a significant effect on ON αRGC cells. Significant difference: **P < 0.01, *P < 0.05.

The light-evoked responses in ON αRGCs did not significantly change after applying 100 μM atropine (the spike frequency after normalization changed from 1 to 0.94 ± 0.18, mean ± SEM, *n* = 5, *p* = 0.76). However, the application of 300 μM atropine decreased the spike frequency significantly in the ON αRGCs (0.57 ± 0.1, *p* = 0.048). The light-evoked response almost disappeared when the concentration of atropine was increased to 500 μM (0.22 ± 0.08, *p* = 0.001). Even after washing for 5 min, the spike frequencies of the ON αRGCs were partially recovered (0.37 ± 0.02, *p* = 0.001; Figures 3A–E).

Similarly, in the OFF αRGCs, 100 μM atropine application had no apparent effect on the light-evoked responses (the spike frequency after normalization changed from 1 to 1.01 ± 0.12, *n* = 7, *p* = 0.93) but increased the spike frequency of background (increased by 0.35 ± 0.19, *n* = 7, *p* = 0.004). After increasing the concentration of atropine to 300 μM, the spike frequency decreased (0.81 ± 0.17, *p* = 0.33). Noticeably, after exposure to 300 μM, 71% of the OFF αRGCs (five of seven cells) had an ON response. The light response of the ON αRGC almost disappeared after the application of 500 μM atropine (0.34 ± 0.04, *p* = 0.004). The spike frequency of the OFF αRGC was recovered after 5-min washing (1.05 ± 0.11, *p* = 0.69; Figures 3F–J). The time latency of light-induced spikes had no change in both ON and OFF αRGCs as the concentrations of atropine increased from 100 to 500 μM. In brief, atropine at a concentration below 100 μM had no significant effects on the light-evoked responses of the ON and OFF αRGCs (Figure 3K).

#### Time- and Concentration-Dependent Effects of Atropine on the Light-Evoked Responses of ON and OFF αRGCs

**Figure 4:**
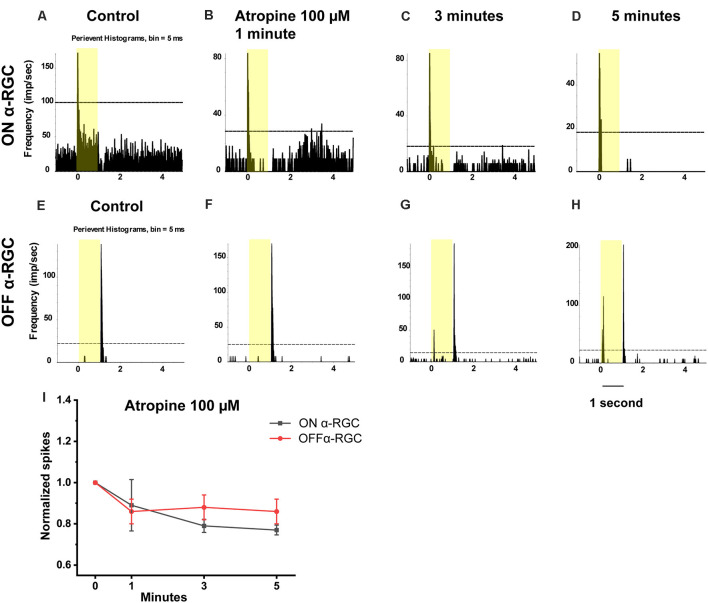
Effect of 100 μM atropine on the light-evoked responses in ON and OFF αRGCs. PSTH showing the light-evoked responses of ON αRGCs [presentation of the 525-nm full-field light stimulation; intensity = 131 Rh* per rod s^−1^ is indicated by the yellow bar; **(A)**]. The spike frequency of light-induced responses of ON αRGCs remained unchanged after exposure to 100 μM atropine from 1–5 min **(B–D)**. PSTH showing the light-evoked responses of OFF αRGCs **(E)**. The spike frequency of OFF αRGCs did not have a noticeable change after 100 μM atropine application from 1 to 5 min **(F–H)**. Notably, an ON response was induced after 3 min **(G)** and persisted for at least 5 min after exposure to 100 μM atropine **(H)**. A graph summarizing the normalized spikes of ON and OFF αRGCs after the application of 100 μM atropine **(I)**. Five-minute exposure to 100 μM atropine had no significant effects on the spike frequencies in both ON and OFF αRGCs.

In this experiment, the time-dependent effects of atropine exposure were examined across a wide range of concentrations. The light-evoked responses (spike frequency) of both ON and OFF αRGCs were recorded following exposure to 100 μM atropine. The spike frequency of the RGCs had no significant difference after 1 min (ON αRGCs: 0.89 ± 0.12, *n* = 5, *p* = 0.42; OFF αRGC: 0.86 ± 0.06, *n* = 9, *p* = 0.06), 3 min (ON αRGCs: 0.79 ± 0.03, *p* = 0.10; OFF αRGCs: 0.88 ± 0.06, *p* = 0.06), and 5 min (ON αRGCs: 0.77 ± 0.02, *p* = 0.09; OFF αRGCs: 0.86 ± 0.08, *p* = 0.06) of exposure to 100 μM atropine. Interestingly, an ON response was induced in 78% (seven of nine) OFF αRGCs 3 min after exposure to 100 μM atropine (Figure 4).

**Figure 5:**
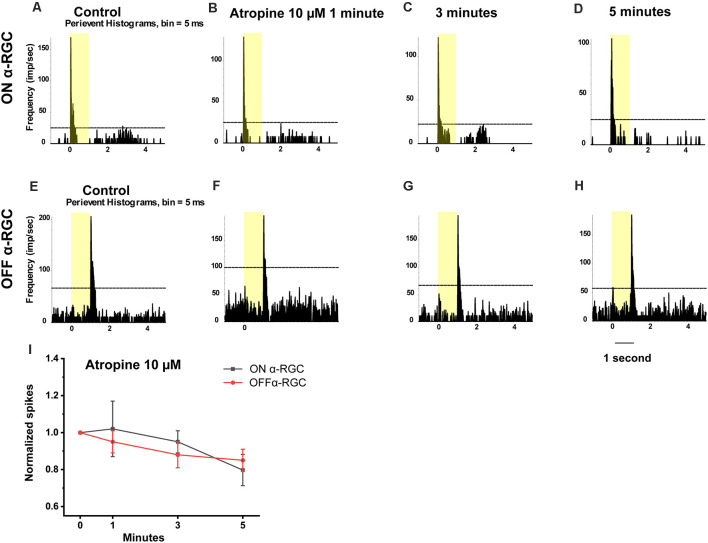
Effect of 10 μM atropine on the light-evoked responses in ON and OFF αRGCs. PSTH showing the light-evoked responses of ON αRGCs [presentation of the 525-nm full-field light stimulation, intensity = 131 Rh* per rod s^−1^ is indicated by the yellow bar; **(A)**]. The spike frequency of ON αRGCs did not change significantly after exposure to 10 μM atropine for 1–5 min **(B–D)**. PSTH showing the light-evoked responses of OFF αRGCs to light **(E)**. The spike frequency of OFF αRGCs did not differ significantly after exposure to 10 μM atropine for 1 to 5 min **(F–H)**. The ON light response was induced 5 min after exposure to 10 μM atropine **(H)**. A graph summarizing the normalized spikes of ON and OFF αRGCs after the application of 10 μM atropine **(I)**. No significant difference in the spike frequency of ON and OFF αRGC was found after exposure to 10 μM atropine.

Then, to test whether the spike frequency was also not significantly affected up to 100 μM, the light-evoked responses of the ON and OFF αRGC were tested following exposure to decreasing concentrations of atropine from 10–0.05 μM in every one log unit step. There was no significant change in the spike frequency of the ON and OFF αRGCs after 1 min (ON αRGCs: 1.02 ± 0.15, *n* = 6, *p* = 0.9; OFF αRGCs: 0.95 ± 0.06, *n* = 8, *p* = 0.44), 3 min (ON αRGCs: 0.95 ± 0.06, *p* = 0.47; OFF αRGCs: 0.88 ± 0.07, *p* = 0.14), and even 5 min (ON αRGCs: 0.85 ± 0.06, *p* = 0.06; OFF αRGCs: 0.88 ± 0.06, *p* = 0.13) of exposure to 10 μM atropine. However, an ON response was induced in 62.5% of all OFF αRGCs (five of eight) after a 3-min exposure to 10 μM atropine (Figure 5).

**Figure 6:**
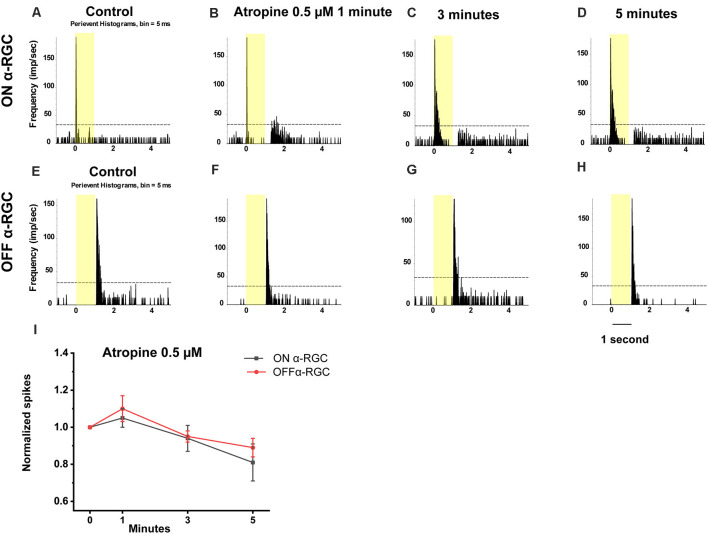
Effect of 0.5 μM atropine on the light-evoked responses of ON and OFF αRGCs. PSTH showing the light-evoked responses of ON αRGCs [presentation of the 525-nm full-field light stimulation; intensity = 131 Rh* per rod s^−1^ is indicated by the yellow bar; **(A)**]. The spike frequency of light-evoked responses of ON αRGCs did not change significantly after exposure to 0.5 μM atropine for 1 to 5 min **(B–D)**. PSTH showing the light-evoked responses of OFF αRGCs **(E)**. The spike frequency of OFF αRGCs did not change after exposure to 0.5 μM atropine from 1 to 5 min **(F–H)**. The ON response was not induced in OFF αRGCs after exposure to 0.5 μM atropine for 5 min. A graph summarizing the normalized spike frequencies of ON and OFF αRGCs showed no significant difference after the application of 0.5 μM atropine **(I)**.

**Figure 7:**
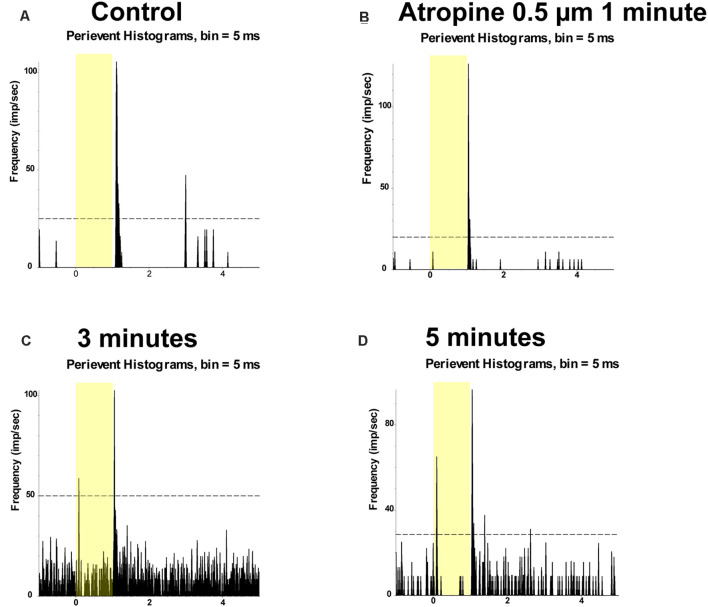
Application of 0.5 μM atropine induced ON responses of OFF αRGCs. PSTH showing the light-evoked responses of OFF αRGCs (presentation of the 525-nm full-field light stimulation; intensity = 131 Rh* per rod s^−1^ is indicated by the yellow bar) **(A)**. The spike frequency of light-induced responses of OFF αRGCs did not change after exposure to 0.5 μM atropine for 1 min **(B)**. However, the ON responses of OFF αRGCs were induced 3 min later **(C)**. The induced ON responses of OFF αRGCs still existed 5 min later **(D)**.

Application of 0.5 μM atropine had even less effects on the spike frequency of the ON and OFF αRGCs after 1 min (ON αRGCs: 1.05 ± 0.05, *n* = 7, *p* = 0.27; OFF αRGCs: 1.1 ± 0.07, *n* = 6, *p* = 0.19), 3 min (ON αRGCs: 0.94 ± 0.07, *p* = 0.42; OFF αRGCs: 0.95 ± 0.03, *p* = 0.1), and 5 min (ON αRGCs: 0.81 ± 0.1, *p* = 0.11; OFF αRGCs: 0.89 ± 0.05, *p* = 0.28; Figure 6). Furthermore, 33.3% of the OFF αRGCs (two of six) showed an ON response after a 5-min exposure to 0.5 μM atropine (Figure 7).

**Figure 8:**
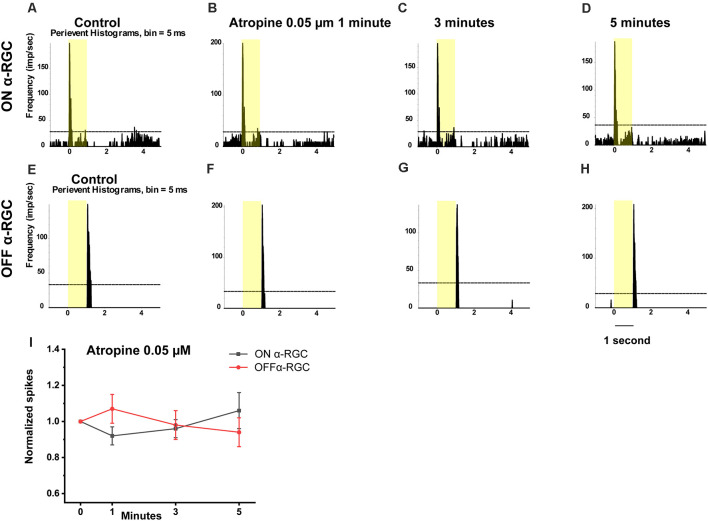
Effect of 0.05 μM atropine on the light-evoked responses in ON and OFF αRGCs. PSTH showing the light-evoked responses of ON αRGCs [presentation of the 525-nm full-field light stimulation; intensity = 131 Rh* per rod s^−1^ is indicated by the yellow bar; **(A)**]. The spike frequency of light-induced responses ON αRGCs did not differ significantly after exposure to 0.05 μM atropine from 1–5 min **(B–D)**. PSTH showing the light-evoked responses of OFF αRGCs **(E)**. The spike frequency of OFF αRGCs did not differ after exposure to 0.05 μM atropine for 5 min **(F–H)**. The ON response was not induced in these cells 5 min later. A graph summarizing the normalized spikes of ON and OFF αRGCs showed no significant difference in spike frequency between the two groups of cells after the application of 0.05 μM atropine **(I)**.

Application of 0.05 μM atropine had no effects on the spike frequency of the ON and OFF αRGCs after 1 min (ON αRGCs: 0.92 ± 0.05, *n* = 5, *p* = 0.27; OFF αRGCs: 1.07 ± 0.08, *n* = 7, *p* = 0.41), 3 min (ON αRGCs: 0.96 ± 0.05, *p* = 0.39; OFF αRGCs: 0.98 ± 0.08, *p* = 0.81), and 5 min (ON αRGCs: 1.06 ± 0.1, *p* = 0.58; OFF αRGCs: 0.9 ± 0.08, *p* = 0.29; Figure 8). Moreover, 28.6% of the OFF αRGCs (two of seven) showed induced ON response after a 5-min exposure to 0.05 μM atropine.

The time latency of the light-induced response of the ON and OFF αRGCs did not change after a 5-min exposure to 0.05–100 μM atropine. In brief, the application of less than 100 μM atropine did not affect the light-evoked response of the ON and OFF αRGCs.

#### Fifty Micromolar Atropine Changed the Joint ISI Distribution of RGCs

**Figure 9:**
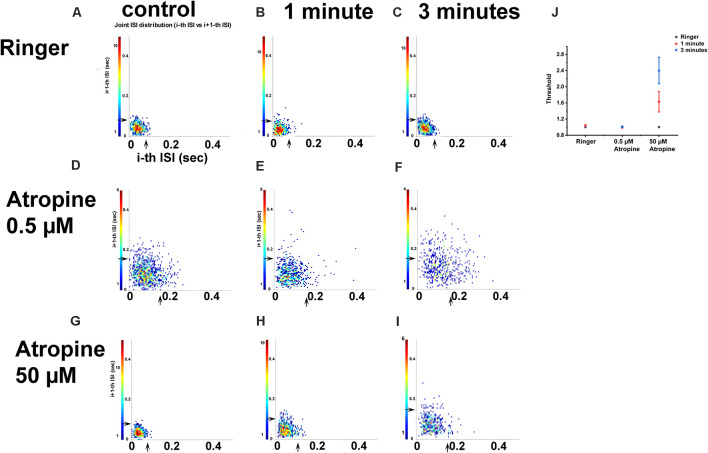
The effect of 0.5 and 50 μM atropine on the inter-spike interval (ISI) distributions of OFF αRGCs. ISI distribution of OFF αRGC spontaneous responses **(A)**. For each spike, a point is plotted, showing the duration of the preceding ISI and the subsequent ISI duration. The arrowhead indicates threshold (T). Cells exposed to Ringer’s solution were used as negative controls (*T* = 0.08 s). The application of Ringer’s solution after 1 min (*T* = 0.08 s; **B**) and 3 min (*T* = 0.08 s; **C**) did not change the inter-spike interval of the cells. The application of 0.5 μM atropine did not affect the joint ISI distribution of the cells **(D–F)**. The thresholds were similar in the control (0.16 s) and the 1 min (0.16 s) and 3 min (0.16 s) setups. The threshold of joint ISI distribution of OFF transient RGC spontaneous responses was 0.08 s **(G–I)**. Application of 50 μM atropine changed the threshold of joint ISI distribution within 1 min (*T* = 0.11 s) and 3 min (T = 0.15 s). A graph summarizing the effect of Ringer’s solution, 0.5 μM atropine, and 50 μM atropine on the threshold of ISI distribution of OFF αRGCs **(J)**. The application of 50 μM atropine had a significant impact on the threshold of ISI distribution of OFF αRGCs.

The spike train pattern of RGCs is defined by the average time of successive ISI over a short time. Joint ISI distribution based on the spike train patterns can be used to identify most retinal cell types (Zeck and Masland, 2007). The concentration-dependent effects of atropine on ISI distribution were tested by recording OFF αRGCs following 1- and 3-min exposures to 0.5 μM atropine under a dim light background (around 1 Rh* per rod/s). The results revealed that the threshold of the joint ISI distribution remained 0.16 s after both time intervals. An increase from 0.5 to 10 μM atropine concentration exposure led to minor changes, which did not reach statistical significance. Exposure to 50 μM atropine led the joint ISI of the cells during spontaneous spikes to increase from 0.08 to 0.11 s after 1-min exposure and a further increase to 0.15 s after a 3-min exposure, which indicated a change in the firing pattern of the cells. Exposure to Ringer’s solution (negative control, *n* = 4) did not change the cells’ joint ISI distribution (Figure 9). The increase in ISI of the αRGCs (*n* = 6) indicated the change of the cells’ firing pattern with 50 μM atropine application. However, the application of low-dose atropine (less than 0.5 μM, *n* = 5) did not affect the signaling of the αRGCs.

#### Low-Dose Atropine Did Not Affect the Synchronized Firing Between OFF αRGCs

Synchronized firing mediated through gap junctions plays an essential role in encoding visual information in the retina (Abbott and Dayan, 1999; Schnitzer and Meister, 2003; Shlens et al., 2008). Synchronized firing between neurons serves to convey more information than independent neuron activity. In this study, Kcng4-YFP mice, in which OFF αRGCs are fluorescently labeled, were used to identify the synchronous patterns between OFF αRGCs. Electrical coupling in the pairing of OFF αRGCs can involve several gap junctional pathways: RGC-RGC, RGC-AC, and AC-AC (Volgyi et al., 2009; Pan et al., 2010). The effect of low-dose atropine on synchronized firing patterns in paired OFF αRGCs was evaluated. To identify synchronized firing activity in OFF αRGC pairs, cross-correlogram profiles for spikes were generated to reveal activity correlation exceeding chance at the 99% confidence level. First, the light-evoked responses of the OFF αRGCs were recorded. To demonstrate spike correlations between the OFF αRGC pairs that were not time-locked to the light stimulus, data were time-shuffled using a shift-predictor protocol, which was then subtracted from the original cross-correlogram. The shift-predicted spikes showed pairs of coupled αRGCs.

**Figure 10:**
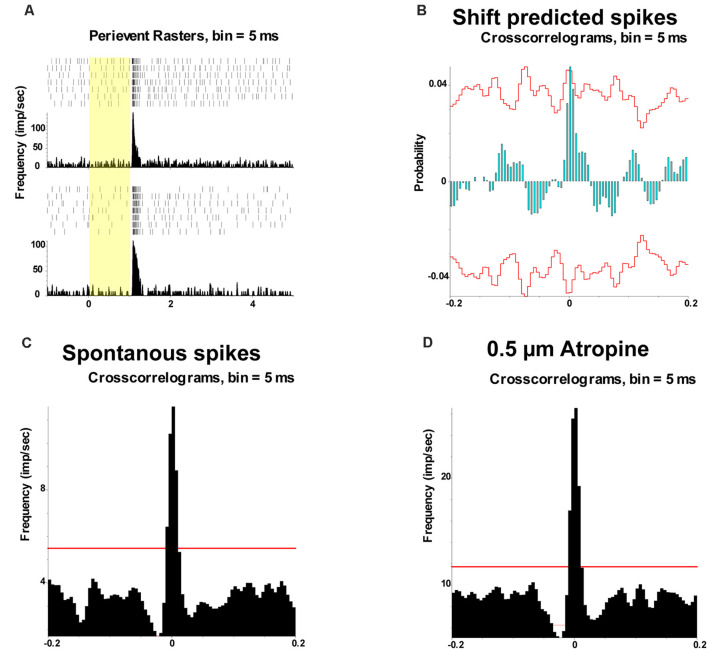
Effect of 0.5 μM atropine on the synchronized firing pattern of RGCs. **(A)** PSTH and perievent raster showing the light-evoked responses of two OFF αRGCs. **(B)** Shift-predicted synchronized firing pattern showing the coupling between the two OFF RGCs **(C)**. Synchronized firing also existed between the spontaneous spikes of the two paired OFF αRGCs **(D)**. Moreover, 0.5 μM atropine did not affect the spontaneous synchronized firing. The width of the synchrony time remained 0.036 ms after atropine application. The red line represents 99% confidence interval.

Spontaneous spike trains were then recorded from pairs of mouse αRGCs. Cross-correlation function (CCF) fit with a Gaussian function was applied to analyze the synchronized spontaneous firing. The unimodal CCFs showed the synchronized firing from the two OFF αRGCs. Application of 0.5 μM atropine did not change the synchronized firing pattern (width = 0.036 ms before and after), indicating that low-dose atropine did not affect the synchronized firings between the coupled OFF αRGCs (Figure 10).

### Discussion

Myopia prevalence is climbing worldwide, particularly in Asian populations. Complications of myopia are associated with substantial economic and social costs. Atropine sulfate is an antimuscarinic agent used as a cycloplegic and mydriatic eye drop. Although clinical trials have shown that low-dose atropine eye drops (0.05, 0.025, and 0.01%) reduce myopia progression (Yam et al., 2019), the impact of atropine on the retina, especially the signaling of RGCs, remains unknown.

#### Application of Low-Dose Atropine Had No Noticeable Effects on αRGCs

Animal experiments have confirmed that atropine sulfate in eye drops reached the retina *via* transcorneal or transconjunctival–scleral pathways. The concentration of atropine in the vitreous humor of the rabbit was approximately 0.4 μM after a 24-h exposure to 1% atropine (Wang et al., 2019). In this study, following immersion of the corneal surface of murine enucleated eyeballs in Ringer’s solution containing 800 μM atropine sulfate (equivalent to 0.05% atropine solution) for 30 min to mimic topical application, the retinal atropine concentration reached 2 uM. The tear fluid has been shown to wash away almost 90–95% of the clinical application of 0.05% atropine eye drop within seconds/minutes. The topical eye drop solution would not be present in the conjunctival sac after 30 min (Hollingsworth et al., 1992). The concentration of atropine reaching the retina is estimated to be 1,000-fold (three log units) lower than that in the applied atropine eye drop, assuming that the blood–ocular and the blood–retina barriers are equally tight across mammalian species. The concentration of atropine used for myopia control varied from 0.01 to 1% (Gong et al., 2017). It is estimated that the concentration of atropine reaching the retina will be varied from 0.05 to 5 μM. In some extreme cases with the blood–ocular and the blood–retina barriers damaged, for example, in trauma and postoperative patients, if the 0.05% atropine was accidentally applied, the concentration of atropine reaching the retina will be up to 500 μM. Hence, when characterizing the effects of atropine exposure directly on the retina, this study tested concentrations as low as 0.05 μM (up to five log units lower) and up to 500 μM.

The mouse eyes are different from the human eyeball in terms of the properties of the blood–ocular and the blood–retina barriers. There is a relatively bigger lens in mouse (average of 2.7 mm in the length of the eyeball, with 1.3 mm in the thickness of lens) but a larger vitreous body in the human eyeball (the human eye is 26.5 mm in length, and the vitreous chamber is about 16.5 mm in axial diameter that occupies a volume of about 4.5 ml; Zhou et al., 2007; Sharif-Kashani et al., 2011). Therefore, the concentration and duration of atropine that reached the retina will be different in the mouse and human eye, even with the same concentration of topical atropine applied. It was estimated that the atropine should be lower in concentration and stay longer within the human retina than in the mouse retina because of the large volume of the vitreous body. Thus, it is safe to apply low-dose atropine in humans.

ACh receptor activation has been shown to affect the light responses of RGCs (Baldridge, 1996; Kittila and Massey, 1997) and retinal development (Zhou and Zhao, 2000). There are two classes of ACh receptors in the retina (Smith et al., 2014): muscarinic ACh receptors (mAChRs) and nicotinic ACh receptors (nAChRs). There are five subtypes of mAChRs, m1–m5, which are G-protein-coupled receptors. In general, the m1, m3, and m5 subtypes all activate the Gq-α G-protein to depolarize (excite) the cell. The m2 and m4 subtypes activate the Gi-α G-protein to hyperpolarize (inhibit) the cells (Brown et al., 1997; Wess et al., 1997). Strang and colleagues performed detailed research about the muscarinic cholinergic system and showed atropine’s effect on various properties of RGCs by blocking muscarinic ACh receptors in the rabbit retina (Strang et al., 2010, 2015). It is possible that high-dose atropine blocked the m1 to m5 receptors differently and drove the mixed effects of excitation and inhibition of RGC light responses to favor more inhibition.

Atropine below 100 μM did not change the spike frequencies of the ON and OFF αRGCs, while 100 μM atropine increased the spike frequencies of OFF αRGCs over time. The spike frequencies of both ON and OFF αRGCs did not change after exposure to <0.5 μM atropine, which is equivalent to the estimated atropine concentration reaching the retina after external ocular application of 1% atropine eye drops. Thus, it is reasonable to conclude that the application of eye drops with concentrations below 1% atropine is unlikely to affect the signaling of ON and OFF αRGCs. This conclusion was further confirmed by the synchronized firing and joint ISI distribution of αRGCs in the retina.

Synchronized firing codes elementary visual information in the retina. More than 20% of visual information may be carried to the brain by synchronized firing (Pillow et al., 2008; Shlens et al., 2008). The application of 0.5 μM atropine did not change the synchronized firing pattern between the OFF αRGCs, including several gap junctional pathways. This provided further evidence that the ocular application of low-dose atropine is unlikely to change the signaling of RGCs. The joint ISI distribution of RGCs reflects the cell properties used in cell type characterization (Zeck and Masland, 2007). The application of low-dose concentrations of atropine again did not affect the spontaneous firing pattern of αRGCs in this study. The concentration of <0.5 μM (low dose) atropine did not interfere with the signaling of RGCs, which again suggests that a topical application of 1% atropine does not affect the joint ISI distribution of RGCs.

#### Effect of Atropine on Light-Evoked Responses of OFF αRGCs Could Involve the GABAergic Pathway

Atropine is a nonselective, broad muscarinic acetylcholine receptor antagonist. The muscarinic acetylcholine receptors 2 (mAChR2) are expressed by cells of the inner nuclear layer and the IPL of rats (Wasselius et al., 1998) and in primate retinas (Yamada et al., 2003). In addition, mAChR2 is also localized in the ganglion cell layer (Strang et al., 2010). In the rabbit retina, mAChR2 immunoreactive amacrine cells also express γ-aminobutyric acid (GABA) receptors and may provide inhibitory feedback to cholinergic amacrine cells (Strang et al., 2010). Thus, ACh could induce GABA release onto rod bipolar cells through heteromeric nicotinic receptors expressed in A17 amacrine cells (Elgueta et al., 2015). The GABAergic pathway in the retina plays a role in the modulation of eye growth and refractive development in animals (Upadhyay and Beuerman, 2020). Research (Barathi et al., 2014) showed that atropine attenuated GABA transporter expression (GAT-1) by the relative quantification of retina proteome in the mouse retina. The application of atropine could block GABA release to unmask inhibition in the inner retina (Ackert et al., 2009; Farajian et al., 2011; Pan et al., 2016; Khanal et al., 2019; Wang et al., 2020). In this study, ON response was unmasked in 78% OFF αRGCs after an exposure to 100 μM atropine. Interestingly, 33.5% OFF αRGCs had ON response after exposure to 0.5 μM atropine, and one-fourth (28.6%) of OFF αRGCs showed ON response after exposure to 0.05 μM atropine. The ON response from OFF αRGC induced by 100 μM atropine can be abolished by puffing 1 mM GABA application (*n* = 5; see the Supplementary Figure 1). The experiment showed that atropine might involve unmasking the GABA inhibition because the ON response disappeared after GABA application. The incidence of a side effect of atropine, especially photophobia, which is related to an increased sensitivity of the retina, climbed with higher concentration. This side effect might relate to unmasking inhibition of the GABAergic pathway, which induced ON response in OFF αRGCs and increased the light sensitivity of the RGCs (Pan et al., 2016).

**Figure 11:**
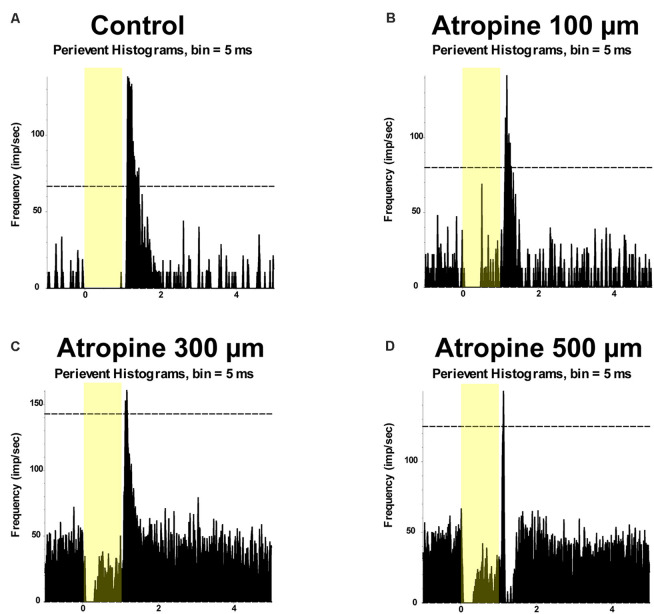
The application of atropine did not induce ON responses in OFF αRGCs of Cx36-knockout (KO) mice. PSTH showing the light-evoked responses of OFF αRGCs in Cx36-knockout mice (presentation of the 525-nm full-field light stimulation; intensity = 131 Rh* per rod s^−1^ is indicated by the yellow bar; **A**). The spike frequency of light-induced responses of the RGC was unchanged after applying 100 μM atropine **(B)**. No ON responses of the αRGC were induced after applying 300 μM atropine **(C)**. No ON responses of the αRGC were induced after applying 500 μM atropine **(D)**.

To further confirm the mechanism, additional experiments needed to be performed. Of note is that ON response was not observed in OFF αRGCs (*n* = 6) of the retinas of homozygous Cx36-knockout mice after up to 500 μM of atropine application (Figure 11). According to our previous report, the OFF αRGCs lost their coupling with amacrine cells in Cx36 KO mice (Pan et al., 2010).

There are around 40 different RGC types in the mouse retina that code distinctive aspects of visual information (Baden et al., 2016). OFF αRGCs are identified based on their distinct morphological and physiological properties, including giant cell bodies, stout dendrites and axons, large mono-stratified dendritic arbors, and light responses (Krieger et al., 2017). OFF αRGCs may include two to four types of OFF RGCs, which have different coupling with amacrine cells and circuits. The reason that not all OFF αRGCs induced an ON response with various concentrations of atropine application may be attributable to the different RGC types. Even if the RGC types were the same, there are also variations in their coupled numbers of amacrine cells, which may differ in their ON response after atropine application induction. It might explain the dose- and time-dependent unmasked ON response induced in OFF αRGCs.

The study revealed that retinal output could change qualitatively, for example, the appearance and disappearance of ON responses in OFF cells across a variety of light stimuli. The results suggest that the retinal code varies with ambient luminance change (Tikidji-Hamburyan et al., 2015). This could be another possible mechanism to induce ON response in the OFF αRGCs after atropine application. Thus, it will be interesting to observe how atropine affected the response of OFF αRGCs across background luminance levels in further experiments.

Gap junctional coupling with amacrine cells mediates crossover excitation in RGCs (Farajian et al., 2011). Cx36 is required for most of the coupling between RGCs and amacrine cells in the mouse retina. Therefore, in this study, Cx36-knockout mice were used as the controls (Pan et al., 2010). ON response was induced in one-fourth of the OFF αRGCs after applying low-dose atropine. This induced ON response in the OFF αRGCs may affect the retina’s encoding and transmission of visual information to the brain.

Low-dose atropine is an effective therapy to control myopia progression. In children, atropine was widely used to prevent myopia progression to high myopia (Shih et al., 1999). The topical atropine varied from 1 to 0.01% in clinical application. Concentrations above 0.5% atropine had a better effect on retarding myopia progression, but with a higher rate of side effects, such as photophobia, which discourages its long-term application. The study in mouse retina showed that the relatively low clinical dosage of topical atropine has no significant morphological and/or physiological effects on the RGCs, but the low-dose atropine (from 0.01 to 0.05%) did induce ON response in at least 25% of OFF αRGCs in the mouse retina. This might relate to the side effect of topical atropine application. Considering that 1 billion people will have high myopia (Holden et al., 2016), the clinical application of atropine should still be with caution, especially in children.

### Conclusion

Low-dose atropine did not change the spike frequency of the ON and OFF αRGCs. However, it may unmask the GABA inhibition, which interfered with retinal signal processing in information transmission to the brain in one-fourth of OFF αRGCs. This study suggests that the topical application of low-dose atropine for myopia control in humans may have unintended consequences on retinal information processing.

### Limitations of the Study

The mouse eye used in the study is tiny compared to the human eye, and the result may not transfer to humans.

In the study, the conclusion that atropine might unmask inhibition to induce ON responses from certain OFF RGCs *via* the GABAergic pathway was based on the preliminary experiments. Further works are needed to confirm whether the GABAergic pathway was involved in the process.

## Data Availability Statement

The raw data supporting the conclusions of this article will be made available by the authors, without undue reservation.

## Ethics Statement

The animal study was reviewed and approved by the Animal Subjects Ethics Sub-Committee of the Hong Kong Polytechnic University (Approval number: 17-18/65-SO-R-OTHERS).

## Author Contributions

QW, SB, CS, CQ, YS, TL, C-HT, and FP contributed to the acquisition, analysis, and interpretation of data. FP contributed to the conception and design of the work and drafting of the article. All authors contributed to the article and approved the submitted version.

## Conflict of Interest

The authors declare that the research was conducted in the absence of any commercial or financial relationships that could be construed as a potential conflict of interest.

## Supplementary Material

SUPPLEMENTARY FIGURE 1 The application of γ-aminobutyric acid (GABA) abolished ON responses induced in OFF αRGCs. Peristimulus time histogram (PSTH) showing the light-evoked responses of OFF αRGCs in wild-type mice (presentation of the 525-nm full-field light stimulation; intensity = 131 Rh* per rod s^−1^ is indicated by the yellow bar). The spike frequency of light-induced responses of an OFF αRGC (**A**). ON responses (arrowhead) of the OFF αRGC were induced after 100 μM atropine application (**B**). ON responses from OFF αRGC were abolished by 1 mM GABA application (**C**).

## Abbreviations

ChAT: choline acetyltransferase
ISI: inter-spike interval
LC-MS/MS: liquid chromatography–tandem mass spectrometry
RGCs: retinal ganglion cells
SEM: standard error of the mean
SD: standard deviation
WT: wild-type.
