# Supplementary figures and images for: The Effect of Low-Dose Atropine on Alpha Ganglion Cell Signaling in the Mouse Retina
Source: Front Cell Neurosci. 2021 May 5;15:664491. doi: 10.3389/fncel.2021.664491 (PMC8131517; doi:10.3389/fncel.2021.664491)

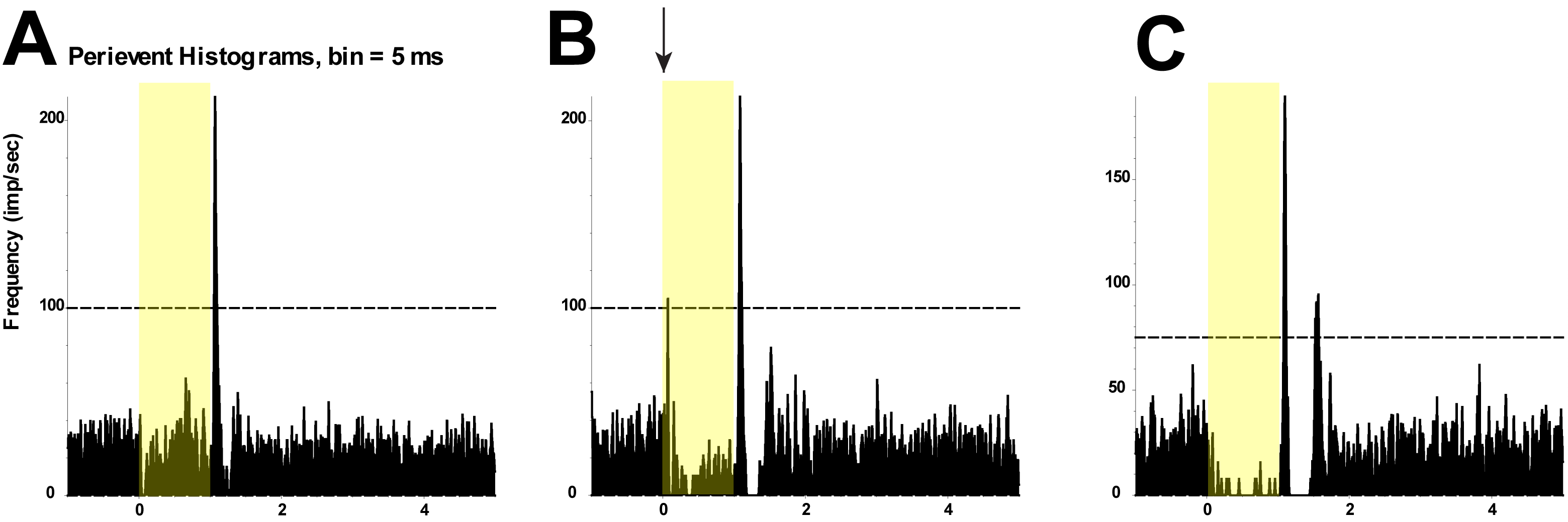

Supplement: SUPPLEMENTARY FIGURE 1 — The application of γ-aminobutyric acid (GABA) abolished ON responses induced in OFF αRGCs. Peristimulus time histogram (PSTH) showing the light-evoked responses of OFF αRGCs in wild-type mice (presentation of the 525-nm full-field light stimulation; intensity = 131 Rh* per rod s−1 is indicated by the yellow bar). The spike frequency of light-induced responses of an OFF αRGC (A). ON responses (arrowhead) of the OFF αRGC were induced after 100 μM atropine application (B). ON responses from OFF αRGC were abolished by 1 mM GABA application (C). [file Image_1.TIF]
